# Preservation of replaced left hepatic artery during 3D laparoscopic totally minimally invasive esophagectomy for cancer

**DOI:** 10.1002/ccr3.2310

**Published:** 2019-07-15

**Authors:** Athanasios Syllaios, Spyridon Davakis, Elias Sdralis, Stamatios Petousis, Bruno Lorenzi, Alexandros Charalabopoulos

**Affiliations:** ^1^ First Department of Surgery Laiko General Hospital, National and Kapodistrian University of Athens Athens Greece; ^2^ Regional Oesophago‐Gastric Cancer Centre, Department of Upper Gastrointestinal Surgery Broomfield Hospital, Mid Essex Hospital Services NHS Trust Chelmsford UK

**Keywords:** 3D, celiac trunk, lymphadenectomy, minimally invasive esophagectomy, replaced left hepatic artery

## Abstract

Preserving a replaced left hepatic artery may be feasible and safe during 3D laparoscopic totally minimally invasive esophagectomy. Avoidance of conversion to an open procedure may be achieved after careful dissection of the celiac trunk lymph nodes, expertise and the visual advantage that 3D vision offers.

## 1 INTRODUCTION

Preserving a replaced left hepatic artery may be feasible and safe during 3D laparoscopic totally minimally invasive esophagectomy. Avoidance of conversion to an open procedure may be achieved after careful dissection of the celiac trunk lymph nodes, expertise and the visual advantage that 3D vision offers.

## 2 CLINICAL IMAGE

**Figure 1:**
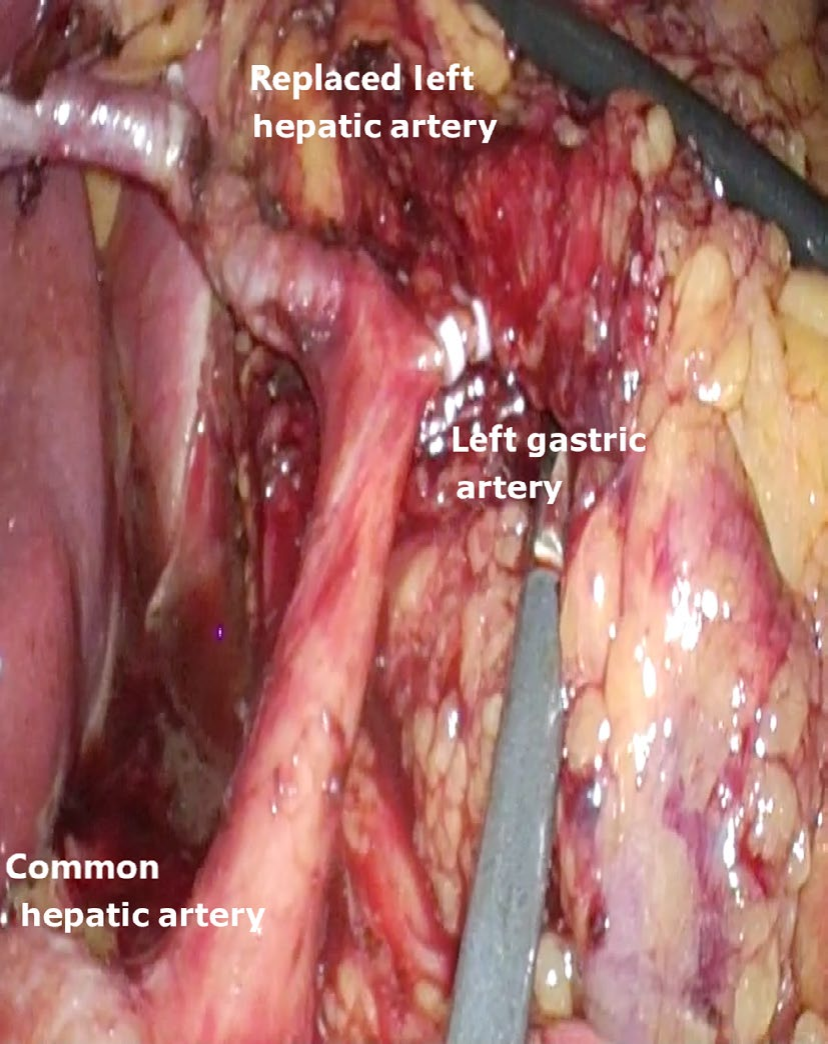
Replaced left hepatic artery originating from the left gastric artery during celiac trunk lymphadenectomy in 3D laparoscopic totally minimally invasive esophagectomy for cancer

A 63‐year‐old‐man diagnosed with midesophageal cancer presented to the Surgical Outpatient Department for surgical treatment. The patient had previously undergone neoadjuvant chemotherapy, upper gastrointestinal tract endoscopy, abdominal and thorax computed tomography (CT), and PET‐CT and was scheduled for a 3‐stage 3D totally minimally invasive esophagectomy (thoracoscopy‐laparoscopy) with 3‐field lymphadenectomy. During the abdominal stage and the D2 abdominal lymphadenectomy, with the patient in supine position, following inspection, during insertion of the lesser sac and dissection of the gastrohepatic ligament, a replaced left hepatic artery (RLHA) originating from the left gastric artery during celiac trunk lymphadenectomy clearance was identified. Consideration to sparing the artery was given. After careful dissection of the celiac trunk lymph nodes, left gastric artery and left hepatic artery were skeletonized and gastric branches of left gastric artery were finally ligated with haemolock clips preserving the replaced left hepatic artery (Figure 1). The stomach was suitable as conduit, and restoration of the alimentary tract was completed. There was no need for conversion to an open procedure, and no postoperative complications or liver dysfunction was observed.

Replaced left hepatic artery is an aberrant vascular formation with a reported incidence of 2.5%‐10%. Finding this abnormality in esophagectomy is relatively rare with an incidence of approximately 5%.^1^ Preoperative abdominal computed tomography CT) has an accuracy of 49%‐67% in detection of a replaced left hepatic artery.^2^ In our case, preoperative identification of that was not feasible; thus, high suspicion from Upper GI surgeons performing minimally invasive esophagectomies to detect this abnormality intraoperatively is urged, in order to avoid possible complications from liver ischemia and liver dysfunction. Preserving a replaced left hepatic artery and obliterating possible tremendous consequences from damaging of this vessel may be feasible and safe during 3D laparoscopic totally minimally invasive esophagectomy. Avoidance of conversion to an open procedure may be achieved after careful dissection of the celiac trunk lymph nodes, expertise and the visual advantage that 3D vision offers.

## CONFLICT OF INTEREST

The authors declare no conflict of interest.

## AUTHOR CONTRIBUTIONS

AS and SD: collected the data, wrote the manuscript, and revised the manuscript. ES,SP and BL: collected the data and revised the manuscript. AC: provided intellectual input and supervised the manuscript.
